# Large-scale epidemiological and diagnostic assessment of canine cytology in Portugal: insights from 12,671 retrospective cases

**DOI:** 10.14202/vetworld.2025.2955-2968

**Published:** 2025-10-08

**Authors:** Paula Brilhante-Simões, Ricardo Lopes, Leonor Delgado, Ana Machado, Augusto Silva, Carla Gomes, Ângela Martins, Ricardo Marcos, Felisbina Queiroga, Justina Prada

**Affiliations:** 1 INNO Veterinary Laboratories, R. Cândido de Sousa 15, 4710–300 Braga, Portugal; 2 Department of Veterinary and Animal Sciences, University Institute of Health Sciences (IUCS), Polytechnic and University Higher Education Cooperative (CESPU), 4585-116, Gandra, Portugal; 3 Department of Veterinary Sciences, University of Trás–os–Montes e Alto Douro (UTAD), 5000-801, Vila Real, Portugal; 4 UNIPRO-Oral Pathology and Rehabilitation Research Unit, University Institute of Health Sciences- Polytechnic and University Higher Education Cooperative (CESPU), 4585-116, Gandra; 5 Animal Health Ireland, 2-5 The Archways, Carrick-on-Shannon, Co. Leitrim N41 WN27, Republic of Ireland; 6 Animal and Veterinary Research Centre (CECAV), Associate Laboratory for Animal and Veterinary Sciences (AL4AnimalS), University of Trás-os-Montes e Alto Douro (UTAD), 5000-801, Vila Real, Portugal; 7 Cytology and Hematology Diagnostic Services, Laboratory of Histology and Embryology, Department of Microscopy, ICBAS-School of Medicine and Biomedical Sciences, University of Porto, Rua de Jorge Viterbo Ferreira, 228, 4050-313, Porto, Portugal; 8 Animal Morphology and Toxicology Team (CIIMAR). Interdisciplinary Centre of Marine and Environmental Research, University of Porto, Matosinhos, Portugal; 9 Center for the Study of Animal Sciences of the Institute of Sciences, Technologies and Agro-Environment of the University of Porto, CECA-ICETA, University of Porto, 4200-465, Porto, Portugal

**Keywords:** canine cytology, diagnostic yield, epidemiology, neoplasia, retrospective study, veterinary diagnostics

## Abstract

**Background and Aim:**

Cytology is a minimally invasive, rapid, and cost-effective diagnostic tool widely used in veterinary medicine for evaluating a broad range of lesions. While extensively applied in clinical practice, large-scale epidemiological analyses of cytologic findings in canine populations are limited. This study aimed to evaluate the diagnostic performance of cytology in dogs in Portugal, assessing associations between lesion type, anatomical location, and demographic factors, and to identify patterns that can improve diagnostic utility in veterinary practice.

**Materials and Methods:**

A retrospective analysis was conducted on 12,671 cytological samples collected from canine patients between January 2010 and December 2016, submitted by 355 veterinary practices across mainland Portugal. Samples were categorized by anatomical location and diagnostic classification (neoplastic, inflammatory, non-neoplastic/non-inflammatory, and other). Demographic variables (sex, age, and breed) were recorded. Statistical analyses included non-parametric tests, logistic regression, and trend analyses to explore associations between diagnostic outcomes and study variables.

**Results:**

The overall diagnostic yield was 69.03%, with the highest rates in fluid (80.21%), mucosal (77.08%), and glandular (75.78%) samples. Cutaneous/subcutaneous lesions, although the most frequent, had the lowest diagnostic yield (66.12%). Of diagnostic cases, 43.94% were neoplastic, with prevalence increasing significantly with age, from 17.75% in dogs ≤1 year to 54.75% in those >10–15 years (p < 0.001). Females were more likely to present epithelial and mesenchymal lesions, while males more frequently had round cell and melanocytic lesions. Breed analysis revealed no significant predispositions after statistical adjustment. Veterinary hospitals achieved higher diagnostic rates than clinics (p = 0.002). Non-diagnostic samples (30.97%) were primarily attributed to poor sample quality or insufficient cellularity.

**Conclusion:**

Cytology is a valuable first-line diagnostic modality in canine veterinary practice, particularly effective for fluid, mucosal, and glandular lesions. Lesion prevalence varies significantly with age and sex, highlighting the need for tailored diagnostic considerations. Standardizing sample collection and preparation techniques, especially for anatomical sites with lower yields, could further improve diagnostic accuracy. While the absence of histopathological confirmation limits definitive classification, the large dataset and standardized diagnostic review strengthen the reliability of findings. Integrating cytology into routine diagnostics can enhance early disease detection, guide treatment decisions, and support epidemiological surveillance.

## INTRODUCTION

Cytology is a simple, minimally invasive diagnostic technique widely applied in veterinary medicine [1–4]. It is a rapid, cost-effective method that carries minimal risk to the patient [4–6]. Due to its accessibility and efficiency, cytology plays a pivotal role in both veterinary and human medicine for the early detection and classification of lesions. Cellular evaluation can be performed using various techniques, including fine-needle aspiration, non-aspiration, impression smears, swabs, and scrapings [7–10]. The choice of collection technique depends on the operator’s preference and the characteristics of the lesion. Samples for cytological evaluation may be obtained from cutaneous and subcutaneous lesions (e.g., masses, cysts, ulcers, fistulas) as well as from internal organs and body fluids, including effusions, cerebrospinal fluid, urine, and synovial fluid [8].

In clinical practice, cytology is frequently used for the preliminary assessment of inflammatory and neoplastic processes, providing timely information to guide therapeutic decision-making. However, it has inherent limitations, such as the absence of tissue architecture, which necessitates histopathological examination for comprehensive evaluation [8, 10]. Diagnostic accuracy can also be compromised by suboptimal sample quality, inconsistent cellularity, and poor slide preparation [11, 12]. Despite these limitations, cytology remains a highly valuable diagnostic tool, particularly when interpreted alongside clinical and imaging findings. A definitive cytological diagnosis is often attainable [13], and even when results are inconclusive, cytological findings can effectively guide further diagnostic workups [14]. Numerous studies have highlighted its utility in diagnosing diverse conditions, including cutaneous and subcutaneous lesions [4, 15–18], oral lesions [1, 19], internal organ disorders [14, 20–23], lymph node pathology [5, 11, 24–27], and bone marrow abnormalities [28]. In oncology, cytology is particularly valuable for providing preliminary information on tumor classification and malignant potential [5, 17, 20, 29–40].

Although cytology is widely recognized as a rapid, minimally invasive, and cost-effective diagnostic method in veterinary medicine, most published studies have focused on specific lesion types, anatomical sites, or small sample cohorts. Large-scale epidemiological analyses covering diverse lesion categories, anatomical distributions, and demographic factors in canine populations are scarce, particularly at a national level. In Portugal, no comprehensive study has yet evaluated cytological submissions from a wide geographical distribution to assess diagnostic yield, lesion prevalence, and associated demographic patterns in dogs. Furthermore, previous reports often lack a systematic comparison of diagnostic success rates across anatomical sites, veterinary clinical settings, and patient characteristics such as age, sex, and breed. This gap limits the ability to identify factors influencing diagnostic accuracy, to develop targeted strategies for improving sample quality, and to refine the role of cytology in routine veterinary diagnostics. The absence of large datasets also hinders epidemiological surveillance and the development of evidence-based guidelines for integrating cytology into broader diagnostic workflows.

This study aimed to perform a large-scale, retrospective analysis of canine cytology submissions across Portugal over a 7-year period, encompassing 12,671 samples from 355 veterinary practices. Specifically, the objectives were to (1) determine the overall diagnostic yield of cytology and its variation by anatomical site, lesion type, and veterinary service tier; (2) assess the prevalence of neoplastic and non-neoplastic lesions in relation to patient age, sex, and breed; (3) identify anatomical locations and sample types with the highest and lowest diagnostic success rates; and (4) explore demographic and clinical patterns that may inform targeted diagnostic strategies. By addressing these objectives, this study provides the first national-level epidemiological overview of canine cytology in Portugal and contributes evidence to optimize the application of cytology in veterinary practice.

## MATERIALS AND METHODS

### Ethical approval

This study was approved by the Institutional Review Board of INNO Veterinary Laboratories (protocol codes INNO.007 and INNO.0026; approved on September 29, 2021), authorizing the anonymous use of veterinary clinical samples for research purposes. All procedures complied with Portuguese legislation governing the ethical use of animals in scientific research (Decree–Law No. 113/2013 of August 7, 2013), which implements Directive 2010/63/EU of the European Parliament and Council.

### Study period and location

A retrospective cross-sectional study was conducted between January 2010 and December 2016 on canine cytological samples obtained from masses, body fluids, and internal organs submitted to INNO Veterinary Laboratories (Braga, Portugal).

### Study design and sample collection

A total of 12,671 samples were received from 355 veterinary practices, including clinics and hospitals, across mainland Portugal. To avoid pseudoreplication, only the first cytological sample per animal was analyzed, and duplicates were excluded using Clinidata software (version 5.3.25, Maxdata Software, S.A., Portugal).

### Sample classification

Samples requiring additional diagnostic modalities (e.g., cerebrospinal fluid, thoracic/abdominal/pericardial effusions, and bone marrow myelograms) were excluded. Each submission included a laboratory request form detailing breed, sex, age, suspected diagnosis, anatomical sampling site, and requested analyses. Age was classified into seven categories [41, 42]:

- Puppy: ≤1 year
- Young: >1–≤2 years
- Young adult: >2–≤4 years
- Adult: >4–≤7 years
- Mature adult: >7–≤10 years
- Senior: >10–≤15 years
- Geriatric: >15 years.

### Cytological evaluation

#### Anatomical location classification

Samples were categorized into 22 anatomical locations, including adnexal glands, bone, bronchoalveolar/tracheal washes, cutaneous/subcutaneous masses, ear, reproductive tracts, gastrointestinal tract, heart, intra-abdominal and intrathoracic masses, lymph nodes, mammary glands, ocular structures, oral cavity, respiratory tract, spleen, synovial fluid, thyroid/adrenal glands, and urinary tract.

### Anatomical groupings for statistical analysis

Seven broader categories were used:

1. Cutaneous and subcutaneous sites
2. Lymph nodes
3. Glands (mammary, endocrine, pancreas, prostate)
4. Body fluids (e.g., synovial fluid, urine, bronchoalveolar/transtracheal washes)
5. Miscellaneous (e.g., intra-abdominal/intrathoracic masses, bone, testis)
6. Mucous membranes
7. Internal organs.

#### Diagnostic categories

- Diagnostic:
  - Neoplastic: Based on cytomorphological features for epithelial, mesenchymal, round cell, and melanocytic lesions [43].
  - Inflammatory: Classified as suppurative, pyogranulomatous/granulomatous, eosinophilic, lymphocytic, plasmacytic, or unknown origin; lesions were deemed infectious when pathogens were detected [7, 43].
  - Non-neoplastic/non-inflammatory: Included cystic, degenerative, and hemorrhagic lesions; hyperplasia; corticosteroid-induced hepatopathy; extramedullary hematopoiesis; and other non-inflammatory changes [44].
  - Other: Normal samples and vaginal cytology for estrous cycle staging.

- Non-diagnostic
  - Suggestive: Findings indicating a possible diagnosis without definitive confirmation [45].
  - Inconclusive: Low/non-representative cellularity, hemodilution, or poor-quality samples [45].

For statistical purposes, diagnostic samples were further grouped into neoplastic and non-neoplastic categories.

#### Slide preparation and review

Cytological slides were stained using Romanowsky-type stains (Hemacolor, Merck KGaA, Darmstadt, Germany) and evaluated microscopically under standardized, quality-controlled protocols. Two experienced veterinary pathologists (P.B.-S. and J.P.) independently reviewed and confirmed all diagnoses, resolving doubtful cases by consensus to minimize interobserver variability.

### Statistical analysis

Data were retrieved from Clinidata software and processed usingMicrosoft Excel (version 2508, Microsoft Office, Washington, USA), JMP (version 14.3, SAS Institute, Cary, NC, USA), DATAtab (DATAtab: Online Statistics Calculator. DATAtab e.U. Graz, Austria, 2024), andMedCalc (version 20.006, MedCalc Software Ltd, Ostend, Belgium, 2021). Non-parametric tests (binomial, Chi-square, Fisher’s exact) were used to compare category frequencies. The Kruskal–Wallis test with Dunn–Bonferroni *post hoc* analysis was applied for multi-group comparisons. Logistic regression with Tikhonov regularization was used to assess breed predisposition, and the Cochran–Armitage Trend test was employed to evaluate ordered-category trends. Odds ratios (OR) with 95% confidence intervals (CIs) quantified associations between age and neoplasia, using ≤1 year as the reference group.

Veterinary settings were classified as small clinics (<3 veterinarians), medium clinics (4–10 veterinarians), or hospitals (>10 veterinarians), based on Federation of Veterinarians of Europe survey criteria [46].

## RESULTS

### Veterinary clinical settings

Of the total of 12,671 animals included in this study, 163 (1.3%; 95% CI: 1.1%–1.5%) were submitted by small clinics, 7,889 (62.3%; 95% CI: 61.4%–63.1%) by medium clinics, and 4,619 (36.5%; 95% CI: 35.6%–37.3%) by veterinary hospitals.

The Chi-square test of independence revealed a statistically significant association between veterinary clinical settings (small clinics, medium clinics, and veterinary hospitals) and the presence of a conclusive diagnosis (p = 0.002). Hospitals had higher conclusive diagnostic rates than clinics.

Similarly, the Kruskal–Wallis test indicated statistically significant differences in the likelihood of diagnostic outcomes (diagnostic vs. non-diagnostic) across veterinary clinical settings (p = 0.002). *Post hoc* Dunn–Bonferroni comparisons revealed significant differences in diagnostic rates between hospitals and clinics (p = 0.001). Although the comparison between hospitals and small clinics was not statistically significant (p = 0.548), hospitals consistently exhibited the strongest association with diagnostic outcomes, as demonstrated by the observed and expected frequencies. Moreover, hospitals achieved the highest diagnostic rates (mean rank = 6455.92), exceeding those of clinics (mean rank = 6266.21), and small clinics (mean rank = 6315.73).

Regarding neoplastic versus non-neoplastic diagnoses, neither the Chi-square test (p = 0.169) nor the Kruskal–Wallis test (p = 0.169) revealed statistically significant differences across veterinary settings. However, mean rank comparisons indicated that hospitals exhibited a slightly lower likelihood of neoplastic diagnoses compared with clinics and small clinics (hospital: 4540.53, clinic: 4632.27, small clinic: 4646.86).

Table 1 presents the diagnostic and non-diagnostic lesions categorized by veterinary clinical settings in this study during the years 2010–2016.

**Table 1:** Diagnostic and non-diagnostic lesions by veterinary clinical settings.

Lesion	Veterinary clinical settings						Total	

	Small clinic		Clinic		Hospital			
			
	n	% within the settings	n	% within the settings	n	% within the settings	n	%
Non-diagnostic	51	31.29	2530	32.07	1343	29.08	3924	30.97
Diagnostic	112	68.71	5359	67.93	3276	70.92	8747	69.03
Total	163	100	7889	100	4619	100	12671	100

### Diagnostic categorization

A total of 12,671 cytological samples from canine patients were analyzed in this study. Among them, 8,747 samples (69.03%) were deemed diagnostic, while 3,924 (30.97%) were categorized as non-diagnostic. Of the 8,747 diagnostic samples, 3,843 (43.94%) were neoplastic, 2,883 (32.96%) were inflammatory, 1,507 (17.23%) were non-neoplastic/non-inflammatory, and 514 (5.88%) were classified under the other category.

### Diagnostic by anatomical grouping

**Figure 1:**
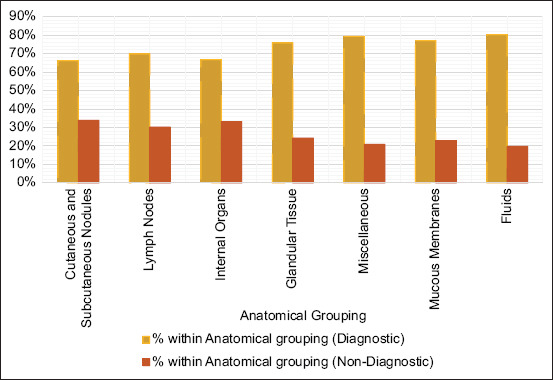
Distribution of diagnostic and non-diagnostic lesions across anatomical groupings.

A significant difference in the distribution of diagnostic and non-diagnostic samples was observed between the anatomical groups (p < 0.001). Cutaneous and subcutaneous nodules comprised the largest category, with 7,573 samples. Fluid samples yielded the highest diagnostic results (80.21%), whereas cutaneous/subcutaneous samples yielded the lowest (66.12%). Figure 1 presents the distribution of diagnostic and non-diagnostic lesions across anatomical groupings in this study.

### Non-neoplastic/neoplastic lesions by anatomical grouping

Of the diagnostic samples, 3,843 (43.94%) were classified as neoplastic and 4,904 (56.06%) as non-neoplastic, highlighting a slightly higher prevalence of non-neoplastic cases. Of the 4,904 non-neoplastic samples, 2,883 (32.96%) were inflammatory and 1,507 (17.23%) were non-inflammatory/non-neoplastic.

A Chi-square test of independence revealed a statistically significant association between anatomical grouping (cutaneous and subcutaneous nodules, lymph nodes, organs, glandular tissue, miscellaneous samples, mucous membranes, and fluids) and the likelihood of neoplasia (p < 0.001).

Cutaneous and subcutaneous nodules represented the largest category, comprising 5,007 samples, with 53.07% classified as neoplastic. Lymph nodes, the second largest group (1,098 samples), exhibited a slightly lower proportion of neoplastic lesions (49.45%). Internal organs and glandular tissue accounted for 620 and 557 samples, respectively, with non-neoplastic lesions predominating in both categories (28.71% and 43.27%, respectively). Miscellaneous samples (560) and mucous membranes (528) had even higher proportions of non-neoplastic lesions (81.96% and 80.11%, respectively). Fluids, the smallest group with 377 samples, exhibited the lowest proportion of neoplastics (4.77%).

**Figure 2:**
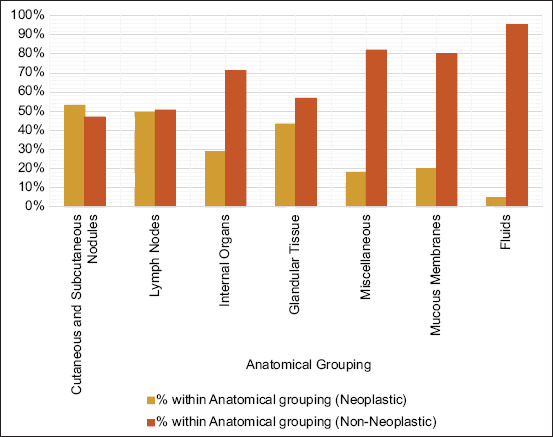
Distribution of neoplastic and non-neoplastic lesions across anatomical groupings.

Figure 2 presents the distribution of neoplastic and non-neoplastic lesions across anatomical groupings in this study.

### Breed

Regarding canine breeds, data were available for 10,939 animals (86.33%); information was lacking in the request form for the remaining 1,732 (13.67%) cases.

A total of 65 different breeds were represented in the dataset, including 3,128 mixed-breed dogs (24.69%), 1,760 Labrador Retrievers (13.89%), 785 Boxers (6.20%), 527 German Shepherds (4.16%), 376 Poodles (2.97%), 358 Cocker Spaniels (2.83%), 345 Golden Retrievers (2.72%), 290 Rottweilers (2.29%), 225 Bulldogs (1.78%), 210 Yorkshire Terriers (1.66%), 202 Siberian Huskies (1.59%), 163 Estrela Mountain Dogs (1.29%), 150 Great Danes (1.18%), 147 Pitbulls (1.16%), 117 Pinchers (0.92%), and 51 other breeds.

For statistical analysis, the canine mixed-breed group was excluded from breed-related statistical analysis to ensure an accurate assessment of breed predispositions [47].

### Non-neoplastic/neoplastic lesions by breed

No significant association between breeds and neoplastic events was noted (Kruskal–Wallis test; p = 0.622). Detailed ranks and pairwise comparisons were conducted, revealing specific breed differences; however, none achieved significance after adjusting for multiple comparisons.

Due to the inconclusive results of these tests, a logistic regression analysis with Tikhonov regularization was conducted to determine if there was a predisposition among various canine breeds to neoplastic events. The results indicated that most breeds did not show a significant association with neoplastic condition (p > 0.05). Among the breeds analyzed, no individual breed consistently showed a predisposition to neoplastic events after adjusting for confounders and multiple comparisons.

### Sex

The canine sexes were distributed as follows: 6,192 (48.9%; 95% CI: 48.0%–49.7%) females and 6,479 (51.1%; 95% CI: 50.3%–52.0%) males. Table 2 presents the respective occurrence of neoplastic events.

**Table 2:** Occurrence of neoplastic events according to sex.

Lesion	Sex				Total	

	Male		Female			
		
	n	% within sex	n	% within sex	n	%
Non-neoplastic	2631	53.65	2273	46.35	4904	52.17
Neoplastic	1878	48.87	1965	51.13	3843	47.83
Total	4509	-	4238	-	8747	100

The Mann–Whitney U test was used to evaluate differences in the frequency of neoplastic events between male and female dogs. The results indicated a statistically significant difference between the groups (U = 9,103,960.5, z = –4.44, p < 0.001), with males exhibiting a lower frequency of neoplastic events compared to females (p < 0.001).

### Non-neoplastic/neoplastic by sex

The distribution of lesion types according to sex was evaluated using a contingency table and the following statistical tests: Chi-square test of independence, Fisher’s exact test, and the Cochran–Armitage Trend test.

The analysis revealed a significant association between sex and neoplastic lesion type (p < 0.001), as demonstrated by the Chi-square test of independence (p < 0.001). Fisher’s exact test confirmed this association, with a two-sided probability (p < 0.001). In addition, the Cochran–Armitage trend test identified a consistent trend in the distribution of neoplastic lesion types between sexes, with statistically significant results (p < 0.001).

Although males showed a higher prevalence of round cell lesions (20.09% in males compared to 17.56% in females) and melanocytic lesions (1.04% in males compared to 0.86% in females), no statistically significant correlation was found between sexes and these specific lesion types (p > 0.05). Females, however, showed a statistically significant association with neoplastic epithelial and mesenchymal lesions (p < 0.001).

Table 3 presents the occurrence of different types of neoplastic lesions categorized by sex.

**Table 3:** Occurrence of different types of neoplastic lesions categorized by sex.

Sex	n (%)	Neoplastic lesions							

		Round cells		Mesenchymal	Epithelial		Melanocytic		Total
				
		% within sex	n (%)	% within sex	n (%)	% within sex	n (%)	% within sex	n (%)
Male	772 (20.09)	41.11	570 (14.83)	30.35	496 (12.91)	26.41	40 (1.04)	2.13	1878 (48.87)
Female	675 (17.56)	34.35	713 (18.55)	36.28	544 (14.16)	27.68	33 (0.86)	1.68	1965 (51.13)
Total	1447 (37.65)		1283 (33.39)		1040 (27.06)		73 (1.90)		3843 (100)

### Age

Information about age was available for 7,754 cases (4,344 [56.02%] non-neoplastic and 3,410 [43.98%] neoplastic).

To investigate whether there was a relationship between age and the development of neoplastic lesions, several statistical tests were conducted, as described below.

The Spearman correlation analysis revealed a significant relationship between age groups and the occurrence of neoplastic lesions. The correlation coefficient (r = 0.22, p < 0.001) indicates a weak but positive correlation, suggesting that the frequency of neoplastic events tends to increase slightly with age.

The distribution of neoplastic lesion types across different age groups was analyzed using a contingency table and Cochran–Armitage trend test. Analysis of the 3,410 cases revealed round cell lesions as the most prevalent overall (37.65%), followed by mesenchymal (33.39%), epithelial (27.06%), and melanocytic lesions (1.90%).

Age-related trends were evident. In younger animals (≤4 years), round cell lesions dominated, comprising 62.86% of lesions in those ≤1 year old and 49.28% in animals aged >1–≤2 years. With increasing age, mesenchymal lesions became more frequent, peaking at 37.73% in the >7–≤10-year age group. Epithelial lesions showed a relatively consistent distribution across all ages, whereas melanocytic lesions remained rare and stable.

The probability of lesions becoming neoplastic clearly increases with age, demonstrating a strong age-related trend. This proportion rose to 23.39% in animals aged >1–≤2 years and further to 30.43% in those aged >2–≤4 years. This trend continued in older animals, with neoplastic proportions reaching 44.65% in the >4–≤7 years group, 49.80% in the >7–≤10 years group, and peaking at 54.75% in the >10–≤15 years group. Even in the oldest animals (>15 years), neoplastic lesions remained prevalent, accounting for 50.36% of cases.

**Figure 3:**
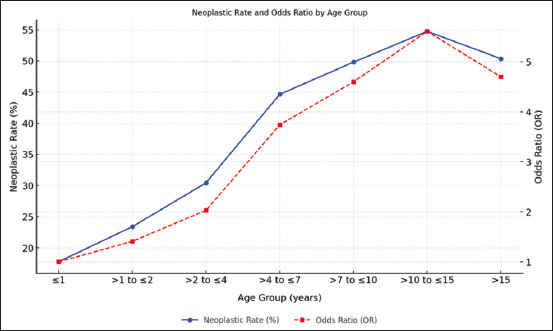
Occurrence of types of non-neoplastic/neoplastic lesions by age group and odds ratios for neoplastic lesions.

The odds of lesions being neoplastic increased with age compared with the reference group (≤1 year). Figure 3 presents the neoplastic lesion rates according to age group and OR.

## DISCUSSION

### Overview of findings

This study conducted a detailed retrospective analysis of cytology as a diagnostic tool in veterinary medicine. Evaluating 12,671 samples from 355 veterinary practices across Portugal, we observed a diagnostic success rate of 69.03%, defined as the proportion of samples yielding a conclusive cytological diagnosis. This highlights the role of cytology in clinical veterinary practice and identifies areas for refinement.

Our findings reinforce the well-established advantages of cytology as a rapid, minimally invasive, and cost-effective diagnostic technique. However, inherent limitations, such as suboptimal sample quality, the absence of tissue architecture, and challenges in achieving a definitive diagnosis, are evident. Notably, 30.97% of the samples were classified as non-diagnostic, consistent with a previous study by Skeldon and Dewhurst [45], which reported an unacceptable sample rate of 19.2%. These results underscore the urgent need for rigorous standardized protocols in sample collection, preparation, and handling to enhance diagnostic reliability.

A limitation of this study was the absence of histopathological confirmation of cytological diagnoses. Although cytology is a valuable diagnostic tool, the lack of histological correlation may reduce the accuracy of specific lesion classifications, particularly in distinguishing between benign and malignant processes. However, the primary aim of this study was not to validate cytologic diagnoses *per se* but to provide a large-scale epidemiological overview of cytologic use and findings in canine patients. It is also important to note that cytology is often not followed by histology in routine clinical settings, especially in patients with inflammatory or non-neoplastic conditions [2–4, 20, 31, 45, 48, 49]. Cytology remains instrumental in diagnosing both neoplastic and non-neoplastic conditions, providing valuable guidance for clinical decision-making. As highlighted in the literature [3], when combined with optimal sample collection techniques and complementary diagnostic modalities, cytology substantially enhances the quality of veterinary care and contributes to improved patient outcomes.

### Veterinary clinical settings

An important finding of this study was the significant disparity in diagnostic outcomes across different veterinary clinical settings. Veterinary hospitals, medium clinics, and small clinics exhibited distinct differences in the proportion of diagnostic versus non-diagnostic samples submitted for cytological evaluation. The statistical analysis revealed a significant association between clinical setting and diagnostic outcomes, highlighting the influence of service tier on the likelihood of obtaining a diagnosis. However, no significant association was found between veterinary clinical settings and the likelihood of neoplastic diagnoses, suggesting that neoplasia is equally distributed among these service tiers.

Veterinary hospitals consistently demonstrated higher diagnostic rates, likely reflecting the availability of advanced diagnostic equipment, in-house expertise, and improved sample quality. These factors contribute to a greater diagnostic yield for complex cases, whereas simpler cases, such as some benign neoplastic lesions (e.g., lipomas and mast cell tumors), are often diagnosed in-house and not submitted for external analysis. This observation is consistent with a previous study by Skeldon and Dewhurst [45], indicating an underrepresentation of routine samples in laboratory datasets. In contrast, medium and small clinics rely more heavily on external laboratories for routine and complex cases.

### Diagnostic yield by anatomical grouping

A significant variation in diagnostic yield across anatomical groupings was observed (p < 0.001), highlighting the influence of the anatomical site on cytological outcomes. Fluids, mucous membranes, and glandular tissues exhibited the highest diagnostic yields (80.21%, 77.08%, and 75.78%, respectively), likely due to their homogeneous cellular composition, which facilitates cytological evaluation [8, 27, 35, 38, 50–53].

In contrast, internal organs demonstrated a lower diagnostic yield of 66.74%, which is consistent with other studies reporting a diagnostic accuracy of 67.39% [20, 23]. This finding may be attributed to the complexity of sampling internal organs and the diverse pathological processes involved.

Cutaneous and subcutaneous nodules constituted the largest proportion of submitted samples, with a diagnostic yield of 66.12%, which is consistent with literature highlighting the effectiveness of cytology in evaluating accessible anatomical locations, reporting diagnostic accuracies of up to 84.5% [15, 34]. Similarly, lymph node samples, with a diagnostic yield of 69.85%, illustrated the utility of cytology in distinguishing reactive, inflammatory, and neoplastic processes, corroborating previous studies by Ku *et al*. [5] and Amores-Fuster *et al*. [11].

The high diagnostic yield in glandular tissues, particularly mammary and endocrine glands, reinforces the value of cytology in detecting both neoplastic and non-neoplastic lesions [1, 38, 54]. Non-diagnostic samples constituted 30.97% of all submissions, with a higher prevalence in cutaneous, subcutaneous, and internal organ samples. These findings highlight the technical challenges posed by these anatomical sites, where factors such as poor sample quality, low cellularity, and hemodilution are more prevalent, reinforcing the importance of optimizing sample collection and preparation techniques to improve diagnostic outcomes.

Future studies should investigate specific factors contributing to lower diagnostic yields at these challenging anatomical locations, such as by comparing different sample collection techniques or evaluating the impact of fixative type on cellular morphology.

### Breed-associated trends

Although breed-related trends in cytological outcomes were observed, no statistically significant association was found between specific breeds and the occurrence of neoplasia. This is in contrast with various studies that identified certain breeds as having a greater predisposition to specific neoplastic events, such as Boxers and Golden Retrievers, recognized in the literature for their predisposition to specific neoplasms, including mast cell tumors and hemangiosarcomas [55–60].

The high proportion of mixed-breed dogs in the study population may have influenced these results, as mixed-breed dogs were excluded from breed-specific statistical analyses. Mixed-breed dogs constituted a substantial proportion of the study population, consistent with their prevalence in the general canine population [61]. Conversely, purebred dogs showed variable representation, with Labrador Retrievers, Boxers, and German Shepherds being the most frequently represented.

The limited sample size for some breeds may have reduced the statistical power to detect breed-specific predispositions. In addition, environmental and lifestyle factors, such as diet, carcinogen exposure, and geographic or socioeconomic influences, may play a more significant role in the development of neoplasia, potentially overshadowing potential genetic predispositions associated with specific breeds [62]. The variability in clinical practices, including differences in diagnostic approaches and sample submission criteria, could also contribute to the observed trends. It is also possible that cytological samples, although providing valuable diagnostic information, may not be ideally suited for detecting subtle breed-specific predispositions to neoplasia.

Future research using larger sample sizes and genetic analysis may be necessary to fully elucidate these relationships.

### Sex-associated patterns

Significant sex-based differences in cytological outcomes were identified, with females exhibiting a higher prevalence of epithelial and mesenchymal lesions, whereas males had a greater frequency of round cell and melanocytic lesions. These findings are consistent with a previous study by Graf *et al*. [63] suggesting that hormonal, genetic, and behavioral factors may influence lesion development.

However, other large case studies have reported contrasting results. For instance, skin neoplasms are more common in males than in females, with epithelial neoplasms being six times more frequent in males [64, 65]. Moreover, other investigations observed no significant differences in the frequency of epithelial and mesenchymal tumors between sexes [58]. These discrepancies may reflect variations in sample populations, study methodologies, or underlying biological factors, underscoring the need for further research to clarify these influences.

Specifically, future studies should investigate the influence of hormonal status (e.g., spayed/neutered status) on the observed sex-based differences in lesion types. Epithelial lesions in female dogs may be linked to hormonal influences, particularly estrogen and progesterone, which play roles in the pathophysiology of various neoplastic and non-neoplastic conditions. For instance, mammary tumors, a frequent finding in intact females, are strongly associated with hormonal influences. Early spaying is well-documented as a protective factor against these tumors, underscoring the clinical importance of identifying sex-related predispositions [66–68].

Conversely, the higher frequency of round cell lesions in males may reflect conditions such as lymphoma and mast cell tumors, which are not directly influenced by sex hormones but may correlate with genetic or environmental factors [69, 70]. The observed male predominance in melanocytic lesions, although not statistically significant, aligns with previous findings in humans and veterinary medicine [71–73].

### Age-associated patterns

A significant correlation was observed between age and the prevalence of neoplastic lesions (p < 0.001), indicating that age plays a crucial role in determining cytological outcomes. The proportion of neoplastic cases increased with advancing age, consistent with literature attributing this trend to cumulative genetic mutations, prolonged environmental exposure, and declining immune function [74, 75].

The cytological findings indicated that adult and senior dogs exhibited a significantly higher prevalence of neoplasia compared to younger cohorts. This observation aligns with existing literature, which attributes the increased risk of neoplastic conditions in older dogs to cumulative genetic mutations, prolonged exposure to environmental carcinogens, and a gradual decline in immune surveillance mechanisms [76, 77].

Chronic inflammatory processes in older animals may also contribute to tumor development, highlighting the multifactorial nature of age-related neoplastic progression. Interestingly, a slight decrease in the OR for neoplastic lesions in animals >15 years may reflect lower susceptibility to neoplasia, likely due to genetic resilience or overall robust health. Moreover, diagnostic priorities in very old animals often shift toward managing clinical symptoms or comorbidities, which may lead to underdiagnosis of neoplastic conditions.

In contrast, younger dogs exhibited a higher prevalence of non-neoplastic lesions, reflecting their predisposition to conditions such as reactive or infectious inflammatory processes, which are less dependent on cumulative genetic and environmental factors [78, 79].

These findings underscore the importance of developing age-specific diagnostic and therapeutic strategies, as the clinical presentation and underlying etiologies vary significantly across different age groups.

### Implications for clinical practice

The results of this study have important implications for clinical practice. The observed variability in diagnostic success across anatomical sites and clinical settings highlights the need for greater standardization of cytology procedures. Implementing training programs on sample collection and preparation, particularly in clinics with lower diagnostic rates, may improve the quality of submissions and reduce the proportion of non-diagnostic cases.

Furthermore, the development and dissemination of standard operating procedures across veterinary facilities could enhance consistency, facilitate inter-clinic comparisons, and ultimately improve patient care.

## CONCLUSION

This large-scale, 7-year retrospective study, encompassing 12,671 cytology samples from 355 veterinary practices across Portugal, demonstrates that cytology remains an indispensable diagnostic tool in veterinary medicine, with an overall diagnostic success rate of 69.03%. The diagnostic yield varied significantly by anatomical site, with the highest rates observed in fluids (80.21%), mucous membranes (77.08%), and glandular tissues (75.78%), and lower rates in cutaneous/subcutaneous lesions (66.12%) and internal organs (66.74%). Veterinary hospitals consistently achieved higher diagnostic success than smaller clinics, highlighting the influence of service tier, equipment availability, and in-house expertise. Age was a significant determinant of neoplasia prevalence, with risk increasing in older dogs, while sex-based patterns suggested hormonal and biological influences on lesion type. Although breed-related trends were observed, no statistically significant predisposition to neoplasia was detected, likely due to the high proportion of mixed-breed dogs and potential environmental or clinical practice factors.

From a practical standpoint, the findings emphasize the importance of cytology as a primary diagnostic tool in veterinary settings, particularly for accessible anatomical sites and initial lesion classification. The results also highlight the urgent need for standardized protocols in sample collection, preparation, and submission, especially in lower-tier clinics, to reduce the 30.97% rate of non-diagnostic samples. Training programs and dissemination of best practices could improve diagnostic consistency and support more timely and cost-effective clinical decision-making.

The strengths of this study include its unprecedented national scope, large dataset, and inclusion of diverse veterinary practice tiers, enabling meaningful epidemiological insights into cytology usage and diagnostic performance. However, limitations include the absence of histopathological confirmation, which may affect the accuracy of lesion classification, and the inability to fully explore genetic predispositions due to sample size constraints in certain breeds.

For future research, integrating cytology datasets with histopathology results would enhance diagnostic validation. Prospective studies comparing sampling techniques, fixation methods, and operator training could directly identify factors influencing diagnostic yield. Expanding the analysis to other species and including molecular diagnostics could further broaden applicability.

This study provides the first comprehensive, nationwide overview of canine cytology in Portugal, reinforcing its clinical value while identifying key areas for procedural refinement. By standardizing practices and leveraging epidemiological insights, the veterinary community can further enhance the diagnostic power of cytology, ultimately improving patient care and outcomes.

## AUTHORS’ CONTRIBUTIONS

PB, FQ, RM, and JP: Conceptualization of the study. PB, RL, LD, AM, and AS: Database processing. PB and RL: Data analysis and drafted the manuscript. PB, RL, CG, and ÂM: Statistical analyses. PB, RL, LD, AM, AS, CG, ÂM, FQ, RM, and JP: Writing–review and editing. FQ, RM, and JP: Designed and supervised the study and reviewed the manuscript. All authors have read and approved the final manuscript.
